# Effectiveness and cost-effectiveness of radiofrequency denervation versus placebo for chronic and moderate to severe low back pain: study protocol for the RADICAL randomised controlled trial

**DOI:** 10.1136/bmjopen-2023-079173

**Published:** 2024-07-26

**Authors:** Kate E Ashton, Cathy Price, Leah Fleming, Ashley W Blom, Lucy Culliford, Rebecca Nicole Evans, Nadine E Foster, William Hollingworth, Catherine Jameson, Nouf Jeynes, Andrew J Moore, Neil Orpen, Cecily Palmer, Barnaby C Reeves, Chris A Rogers, Vikki Wylde

**Affiliations:** 1 Bristol Trials Centre, Bristol Medical School, University of Bristol, Bristol, UK; 2 Solent NHS Trust, Southampton, UK; 3 Faculty of Health, The University of Sheffield, Sheffield, UK; 4 STARS Education and Research Alliance, Surgical Treatment and Rehabilitation Service (STARS), The University of Queensland, Saint Lucia, Queensland, Australia; 5 Population Health Sciences, Bristol Medical School, University of Bristol, Bristol, UK; 6 Musculoskeletal Research Unit, Bristol Medical School, University of Bristol, Bristol, UK; 7 NIHR Bristol Biomedical Research Centre, University Hospitals Bristol and Weston NHS Foundation Trust and University of Bristol, Bristol, UK; 8 BMI Healthcare, The Ridgeway Hospital, Swindon, UK

**Keywords:** Chronic Pain, Pain management, Clinical Trial, Back pain, Quality of Life

## Abstract

**Introduction:**

Low back pain (LBP) is the leading global cause of disability. Patients with moderate to severe LBP who respond positively to a diagnostic medial nerve branch block can be offered radiofrequency denervation (RFD). However, high-quality evidence on the effectiveness of RFD is lacking.

**Methods and analysis:**

RADICAL (RADIofrequenCy denervAtion for Low back pain) is a double-blind, parallel-group, superiority randomised controlled trial. A total of 250 adults listed for RFD will be recruited from approximately 20 National Health Service (NHS) pain and spinal clinics. Recruitment processes will be optimised through qualitative research during a 12-month internal pilot phase. Participants will be randomised in theatre using a 1:1 allocation ratio to RFD or placebo. RFD technique will follow best practice guidelines developed for the trial. Placebo RFD will follow the same protocol, but the electrode tip temperature will not be raised. Participants who do not experience a clinically meaningful improvement in pain 3 months after randomisation will be offered the alternative intervention to the one provided at the outset without disclosing the original allocation. The primary clinical outcome will be pain severity, measured using a pain Numeric Rating Scale, at 3 months after randomisation. Secondary outcomes will be assessed up to 2 years after randomisation and include disability, health-related quality of life, psychological distress, time to pain recovery, satisfaction, adverse events, work outcomes and healthcare utilisation. The primary statistical analyses will be by intention to treat and will follow a prespecified analysis plan. The primary economic evaluation will take an NHS and social services perspective and estimate the discounted cost per quality-adjusted life-year and incremental net benefit of RFD over the 2-year follow-up period.

**Ethics and dissemination:**

Ethics approval was obtained from the London—Fulham Research Ethics Committee (21/LO/0471). Results will be disseminated in open-access publications and plain language summaries.

**Trial registration number:**

ISRCTN16473239.

**STRENGTHS AND LIMITATIONS OF THIS STUDY**

- The trial has a pragmatic design integrated into standard care pathways.
- Guidelines for radiofrequency denervation (RFD) technique were developed during a national workshop with pain clinicians, ensuring that the techniques used in the trial are acceptable to clinicians and reflect best practice recommendations.
- A training video has been developed to support clinicians in performing the RFD technique to be used in the trial.
- Offering participants who do not experience an improvement in pain after 3 months the alternative intervention to which they were randomised may increase trial acceptability while maintaining blinding.
- There is a time lag between consent (waiting list for RFD) and randomisation (in theatre), which may impact on participant engagement.

## Introduction

 Low back pain (LBP) is the leading global cause of healthy life years lost due to disability,^1^ and between 58% and 84% of people in the UK will experience back pain in their lifetime.^2^ LBP is associated with high personal, societal and economic burden.^3^ It can impact many aspects of patients’ lives, and in some cases cause life-changing psychological and social consequences including disengagement from meaningful activities, changed identity, psychological problems, damaged relationships and inability to work.^4 5^ LBP is the most common musculoskeletal reason for general practitioner appointments, accounting for 417 consultations per year per 10 000 patients registered^6^; approximately one-third of the direct healthcare costs associated with LBP are incurred in the hospital sector.^7^

Non-surgical interventions recommended by the National Institute for Health and Care Excellence (NICE) for conservative management of LBP are self-management, exercise, psychological therapy, combined physical and psychological programmes, and non-steroidal anti-inflammatory drugs.^8^ NICE guidelines also recommend that patients with moderate to severe LBP, clinical features suggesting that a facet joint is the main source of pain and insufficient improvement in symptoms with conservative management, can be offered radiofrequency denervation (RFD) of the medial nerve to a facet joint, providing that they have a positive response to a diagnostic, local anaesthetic medial nerve branch block (MNBB). RFD is a minimally invasive outpatient procedure, where a needle is placed into the back and heated up to damage the nerve, thereby interrupting the pain signal. Approximately 13 000 RFDs of the lumbar facet joints are performed annually in the National Health Service (NHS), with a cost to the NHS of around £22 million per year.^9^

Systematic and narrative reviews of the effectiveness of RFD have been published with conflicting conclusions.^10^,^16^ A Cochrane review, published in 2015, concluded that there was no high-quality evidence that RFD provides pain relief for patients with chronic LBP.^15^ In 2017, the MINT trial (published after the systematic reviews) concluded that RFD combined with an exercise programme was not superior to an exercise programme alone.^17^ However, this trial received criticism on a number of methodological grounds, including, variation in RFD operator protocols and high numbers of patients in the control group receiving RFD.^18^,^22^ Hence, the effectiveness of RFD is uncertain due to a lack of high-quality evidence,^15^ and NICE recommends that further research is needed.^8^

The RADICAL (RADIofrequenCy denervAtion for Low back pain) trial aims to provide this evidence by comparing the effectiveness and cost-effectiveness of RFD versus placebo for chronic moderate to severe localised LBP. Specific objectives are to estimate (1) the difference between groups in pain severity 3 months after RFD; (2) differences between groups in back-specific disability, health-related quality of life (HRQoL), psychological distress, time to pain recovery, satisfaction with treatment outcome, frequency of uptake of offer of repeat RFD, adverse events, work outcomes and further healthcare use and (3) the cost-effectiveness of RFD compared with placebo.

## Methods and analysis

### Trial design

**Figure 1:**
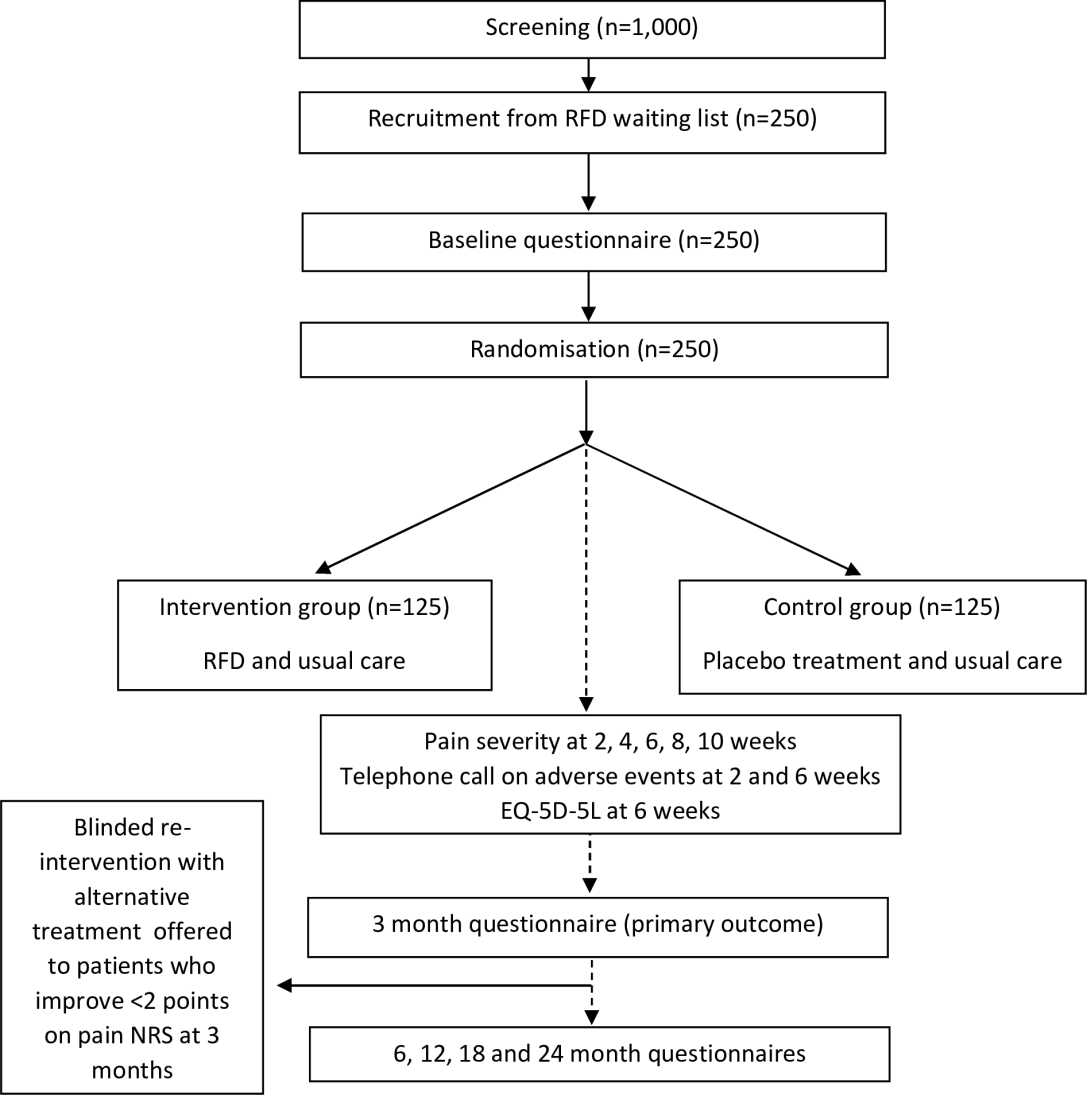
Trial schema. EQ-5D-5L, EuroQol 5-dimension five-level questionnaire; NRS, Numeric Rating Scale; RFD, radiofrequency denervation.

RADICAL is a multicentre, pragmatic, double-blind, parallel-group, placebo-controlled, superiority randomised controlled trial. Patients will be recruited from approximately 20 multidisciplinary pain and spinal clinics providing RFD in secondary care NHS centres (figure 1).

### Eligibility criteria

Patients will be eligible for the study if all the following apply:

1. ≥18 years of age.
2. LBP is the primary source of pain.
3. Positive response to a single diagnostic MNBB with no steroids administered. Based on the outcome of a meeting of RADICAL clinicians,^23^ a positive response is defined as ≥60% pain relief in the first 24 hours, based on patient-reported assessment. Final eligibility will be met if a patient’s pain returns to ≥5 on a 0–10 Numeric Rating Scale (NRS) after MNBB.
4. Chronic LBP (>3 months duration) assumed due to the fact the patient was listed for MNBB.
5. Moderate to severe LBP (NRS score ≥5).
6. Listed for RFD.

Patients will be excluded if any of the following apply:

1. Known pregnancy.
2. Severe depression (Hospital Anxiety and Depression Scale^24^ depression score ≥15) (assessed following consent).
3. Known previous RFD.
4. Known previous back surgery where metal-work has been used in the lumbar spine.
5. Pacemaker or implantable cardioverter defibrillator.
6. Clinical suspicion that an alternative diagnosis is the reason for LBP (as defined by NICE,^8^ including, but not limited to: metastatic spinal cord compression, spinal injury, spondyloarthritis or cancer).
7. Prisoners.
8. Lacks capacity to consent.
9. Existing co-enrolment in another clinical study if (1) the intervention in the other study is expected to influence the primary outcome; (2) it is considered too burdensome for the patient or (3) it is not permitted by the other study.

No restrictions will be placed on usual care, and all co-interventions are permitted to reflect usual NHS practice. Data on co-interventions will not be collected.

### Patient recruitment

Potential patients will be identified from RFD waiting lists and those potentially eligible will receive a patient information leaflet (PIL). The PIL will contain a web address where patients can access an information video to supplement the PIL. The local research team will then contact the patient to discuss the study further and answer any questions they may have. If a patient meets the initial eligibility criteria and decides to participate, the research team will request written informed consent. A copy of the informed consent form can be requested by contacting the RADICAL study team at radical-study@bristol.ac.uk. Eligibility for randomisation will depend on further (post-consent) eligibility checks.

Details of all patients approached and reasons for non-participation will be documented. Participants will also be given the option for their data to be stored for potential use in future research and/or training. Participants can withdraw at any time and will be treated according to standard hospital procedures. Data collected up until the point of withdrawal will be included in the analysis.

### Randomisation and blinding

Randomisation of eligible participants will be performed using a secure internet-based randomisation system, ensuring allocation concealment. Participants will be allocated in a 1:1 ratio to either RFD or placebo. A computer-generated allocation sequence will be prepared by an independent statistician, using random permuted blocks of varying size and stratified by the operator to ensure that any operator effect is distributed equally across groups.

Participants, their clinical care team and the local research team will not be informed of the allocation. Radiofrequency machines to be used in the trial will have to meet key criteria, including having an appropriate method for maintaining blinding of the clinical team and the participant. The trained randomiser will randomise the participant and then control the electrode temperature. The machine display (showing the temperature) will not be visible to the rest of the team in the theatre. This person will have no other role in the trial. Treatment allocation will only be unblinded on participant request or if clinically indicated; for example, in the event of a serious adverse event requiring knowledge of the allocation for treatment. The success of blinding will be assessed using the Bang Blinding Index.^25^

### Intervention

The intervention is RFD of the lumbar medial branches of the dorsal rami performed under local anaesthetic, with sedation if needed. Although there are international consensus practice guidelines for performing RFD,^26^ there is considerable variation in RFD technique across clinicians and centres in the UK.^27^ To refine the RFD technique for the trial, a national consensus meeting was held with clinicians, patients and academics.^23^ Components of the RFD procedure were classified as mandatory or recommended (see online supplemental file), based on existing best practice recommendations.

### Placebo

A placebo control will be used to minimise bias, which is important as the primary outcome is patient reported. The placebo treatment will follow the same RFD protocol as the intervention group, but the temperature of the electrode tip will not be raised.

### Clinical training

Clinicians who are unfamiliar with the RFD technique used within the trial will complete training prior to delivering the trial intervention. This will include an online video (https://www.youtube.com/watch?v=j4nzkdgMWgI) and/or attendance at cadaver workshops.

### Quality assurance measures

X-rays from at least three views for each lesion, from each clinician’s first case, will be shared with a clinical expert on the Trial Management Group (TMG) so that needle placement can be checked. Placement quality will be recorded and feedback will be given. If needle placement is poor, the study clinical experts will agree on a way forward, discuss with the TMG and give feedback to the clinician on a case-by-case basis. X-rays from at least three views for each lesion, for every participant procedure, will be saved locally, for potential future monitoring.

### Adverse events

Adverse events that are expected due to RFD will be recorded between randomisation and 2 weeks post-randomisation. Serious adverse events will be recorded between randomisation and the 2-year follow-up. Between randomisation and 6 months post-randomisation, all unexpected or fatal serious adverse events will be reported to the sponsor.

### Outcomes

The primary outcome is LBP severity (average intensity of LBP over the past week, assessed using the 0–10 NRS) at 3 months post-randomisation. Secondary outcomes will be collected up to 2 years after randomisation and include:

1. Functional disability is measured using the Oswestry Disability Index^28^ version 2.1b.
2. HRQoL was measured using the EuroQol 5-dimension five-level questionnaire (EQ-5D-5L).^29^
3. General health is measured using the 12-Item Short Form Survey (SF-12) Physical Component Score.^30^
4. Mental health is measured using the SF-12 Mental Component Score.
5. Time to pain recovery: time from randomisation until the participant first reports a pain reduction of ≥60% that remains at ≥60% lower than baseline at their next assessment.
6. Uptake of offer of alternative treatment (ie, blinded cross-over to RFD/placebo) after 3 months.
7. Satisfaction with treatment outcome using a Likert scale.
8. Adverse health events.
9. Work outcomes were assessed using the Work Productivity and Activity Impairment (WPAI) questionnaire.^31^
10. Resource use assessed via a patient-reported resource use questionnaire.

### Data collection

Screening data will be collected before consent to establish patient eligibility. Some demographic data (see online supplemental file), information about pain severity and duration of current LBP episode will also be collected from participants and non-participants, as far as possible, at the time of screening, to characterise the population and to interpret the applicability of the trial findings to the reference population. The schedule of data collection outlined in online supplemental table 1 will take place after consent has been received. Data will either be collected on paper data collection forms and entered into the study database or entered directly into the database. Data for the primary outcome and most secondary outcomes will be collected via patient-completed questionnaires. Participants will be followed up at 2, 4, 6, 8 and 10 weeks for pain severity, as well as HRQoL at 6 weeks and adverse health events at 2 and 6 weeks. After this, participants will complete postal/online questionnaires at 3, 6, 12, 18 and 24 months. The participant’s time in the study will end after they have completed the follow-up at 24 months post-randomisation. The end of the study as a whole will be after all participants have completed the follow-up, all data queries have been resolved, the database locked and the analysis completed.

### Sample size

A sample size of 250 participants (125 per group) is sufficient to detect a difference of at least 0.84 in the pain severity NRS (scored 0–10) between randomised groups with 90% power and 5% two-tailed significance, assuming:

- The SD for the pain NRS is 2.0 (17).
- Correlation between NRS at baseline and 3 months is 0.3 (based on data from the MINT trials provided by collaborator Professor Raymond Ostelo).
- Allowing for up to 10% attrition at 3 months.

The trial will, therefore, have sufficient power to detect the target difference used by NICE (1-point difference) and reflect a moderate effect size.^32^

### Statistical analyses

The data will be analysed on an intention-to-treat basis and will follow a prespecified statistical analysis plan.

The primary outcome (NRS) will be analysed using linear mixed effect models, including all available repeated pain measurements up to 3 months, adjusted for time point and the treatment×time point interaction as fixed effects, and operator and participant as random effects. Treatment effects at 3 months will be reported with 95% confidence interval. Protocol deviations will be documented, and a per-protocol secondary analysis will be considered if there are a substantial number of protocol deviators. A secondary responder analysis of the primary outcome will be performed, exploring the between-group difference in the proportion of participants achieving ≥30% improvement in pain from baseline as recommended by the Initiative on Methods, Measurement and Pain Assessment in Clinical Trials,^33 34^ and the number needed to treat will be calculated based on this analysis.^35^,^37^

Continuous and binary secondary outcomes will also be compared using mixed models; and if the treatment×time point interaction is significant at the 10% level, treatment effects at 3, 6, 9, 12, 18 and 24 months will be reported. Time to pain recovery will be analysed using survival methods. Frequencies of adverse events will be described. Missing data on patient questionnaires will be dealt with according to the scoring manuals. Imputation methods, for example, multiple imputation, will be considered if the proportion of missing data is >5%, otherwise complete-case analysis will be undertaken.

Subgroup analyses for the primary outcome will be analysed by adding a treatment by subgroup interaction to the model. Subgroups include younger versus older age (split at median); sex; lower versus higher (split at median) index of multiple deprivation; isolated versus widespread pain; ≥80% reduction in NRS vs ≥60%–79% reduction in NRS in response to the MNBB; low/medium versus high risk of persistent disabling pain based on the STarT Back tool.^38^

Exploratory analyses will assess the effect of reintervention with the alternative treatment using methods developed to appropriately adjust for treatment switching.^39^ Exploratory analyses will also be undertaken to assess the learning effect of the intervention for those less experienced practitioners with fewer than 20 procedures by including procedure numbers in the model. Screening data will be compared descriptively between randomised and non-randomised patients, to ascertain generalisability of results. No formal interim analysis is planned.

### Cost-effectiveness analyses

The analysis will follow a pre-specified health economic analysis Plan. We will use NHS reference costs to estimate the cost to NHS purchasers of RFD. NHS (secondary, primary care, prescriptions), social service, informal care and absenteeism due to LBP will be collected using resource use questionnaires and the WPAI administered to participants throughout follow-up. We will seek consent for data linkage to access Hospital Episode Statistics inpatient, day case, outpatient and emergency department datasets. Hospital, primary and community care will be costed using national unit costs.^40 41^ Quality of life will be assessed using EQ-5D-5L^42^ to calculate quality-adjusted life-years (QALYs). An index score will be derived using the UK value set recommended by NICE at the time of analysis. QALYs will be estimated adjusting for baseline differences in utility scores and any mortality observed during follow-up.

The economic analysis will take an intention-to-treat approach with imputation of missing data (eg, using multiple imputations). In the primary economic analysis, we will estimate the cost per QALY gained of RFD at 2 years from the perspective of NHS and social services. Based on the current NICE willingness to pay thresholds for a QALY of £20 000–£30 000, we will use net benefit regressions, adjusting for baseline EQ-5D-5L scores and baseline characteristics to estimate the incremental net benefit (and 95% confidence interval) and determine whether RFD is a cost-effective use of NHS funds. Uncertainty will be explored using cost-effectiveness acceptability curves. In additional analyses, we will also estimate the cost per QALY gained and cost per additional responder (≥30% improvement in pain) at 3 months and expand the perspective of the analysis to include informal care and productivity costs.

### Internal pilot phase

RADICAL includes a 12-month internal pilot phase with embedded qualitative research. Progression from the pilot to the main study will be contingent on demonstrating that after 12 months of recruitment, enough patients are eligible for the trial and can be randomised. Progression criteria are:

1. 13 sites are open to recruitment.
2. 79 patients consented.
3. 25 patients randomised (this accounts for a 3-month time lag between consent and randomisation).
4. Consent rate of 1.5 patients/site/month.

Qualitative research will be conducted in the internal pilot to evaluate trial acceptability and equipoise and facilitate improvements in communication about the trial to optimise recruitment. Up to 20 recruitment consultations will be audio recorded, and telephone interviews with up to 20 participants will elicit patient understanding of trial procedures and interventions, equipoise, acceptability of recruitment pathways and quality of patient information. Telephone interviews with up to 15 clinicians and 10 recruiters will allow understanding of trial personnel’s equipoise and perspectives on the protocol, usual care and recruitment pathways. Data will be subjected to rapid thematic framework analysis^43 44^ to ensure findings are reported and implemented in a timely fashion.

### Data handling, storage and sharing

Most data will be stored in a bespoke database hosted on the NHS network. Some data items will be held on a separate database, hosted on the University of Bristol server, comprising the randomisation system, information about the intervention delivered and the quality of needle placement. Access to both databases will be via secure password-protected web interfaces.

All study documentation will be retained in a secure location during the conduct of the study and for 5 years afterwards when all participant identifiable paper records will be destroyed by confidential means. All audio recording files will be retained in a secure location during the conduct of the study and for 12 months afterwards when these files will be deleted. Where trial-related information is documented in the medical records, these records will be identified by a label bearing the name and duration of the trial. In compliance with the Medical Research Council Policy on Data Sharing, and with participant agreement, relevant ‘meta’-data about the trial and the full dataset, but without any participant identifiers other than the unique study identifier, will be held indefinitely. These will be retained because of the potential for the raw data to be used subsequently for secondary research and/or training.

### Patient and public involvement

RADICAL was designed in collaboration with a musculoskeletal patient and public involvement (PPI) group at the University of Bristol. A PPI group involving patients with experience of RFD has also been convened specifically for this study. This group has played an integral part in designing the research, including the development of accessible participant documents. They will continue to cowork with the research team on all aspects of the study, including interpretation of results and development of public dissemination strategies and material. The Trial Steering Committee (TSC) also includes two patient members.

### Ethics and dissemination

The study received Research Ethics Committee (REC) approval from London—Fulham REC in July 2021 and Health Research Authority (HRA) approval in September 2021. The study is sponsored by North Bristol NHS Trust (https://www.nbt.nhs.uk/research-innovation) who are responsible for the oversight of the study and ensuring it is managed appropriately. The study is coordinated by the Bristol Trials Centre, a UK Clinical Research Collaboration registered Clinical Trials Unit (Reg. No 70) and overseen by the TSC and a Data Monitoring and Safety Committee (see online supplemental file).

### Changes to the protocol since REC/HRA approval

Following REC and HRA approval the following changes have been made to the study protocol: (1) two amendments to the time frame for assessing response to the MNBB; (2) increase in number of X-ray images to be saved for quality assurance purposes; (3) clarification regarding the mandatory and recommended components of the RFD procedure protocol, to match usual variability in standard practice while still adhering to the same technique and to reflect advances in equipment; (4) muting the sound of the radiofrequency machine (the original proposed method to maintain blinding) was found not to be an option due to safety factors, therefore, it was mandated that sites must have an alternative appropriate solution in place (further details are provided in online supplemental file); (5) telephone calls instead of two-way text messages for assessment of pain severity over the first 10 weeks after randomisation; (6) recruitment pathway shortened so that patients are recruited once listed for RFD rather than after listing for MNBB and (7) added flexibility regarding protocol for MNBB. Protocol version 5.0 (dated 6 April 2023) is currently in use. All relevant parties are informed of protocol amendments.

### Dissemination of findings

Findings will be presented at conferences and published open access in peer-reviewed journals. Impact on clinical practice will be through engagement with relevant organisation such as NICE, British Pain Society, Clinical Reference Group for Spinal Services and UK Spine Societies Board. We will work with our PPI group and relevant charities on public dissemination.

## Discussion

Findings from the RADICAL trial will contribute to shaping clinical guidelines and service provision for patients living with chronic LBP. Study training resources, developed in line with the consensus-based best practice guidelines for RFD produced by the RADICAL team,^23^ have been positively received and taken up by clinicians across the country, demonstrating that the trial is already impacting on RFD provision by improving standards. The study opened to recruitment on 27 May 2022 and is currently recruiting across 17 centres. As of 8 February 2024, 83 patients have been recruited and 47 randomised. The original study end date was 31 December 2024. An extension until 31 July 2026 is currently being requested to complete the study.

During the internal pilot phase, RADICAL experienced three substantial challenges to delivery: delays in site opening, complex screening processes limiting site capacity to recruit patients and long NHS waiting times for RFD. Opening sites has been an ongoing issue due to the continuing impact of the COVID-19 pandemic on research infrastructure; we have experienced delays of up to 2 years from feasibility assessment to site opening due to research and development departments’ limited capacity to process local approvals. However, we have recently seen an improvement in site opening timelines, with a recent site opening in 4 months. Identification of sites with the necessary clinical expertise and engagement, alongside the research infrastructure to deliver the trial, has been key, and we have achieved this through a combination of national calls for sites through the National Institute for Health Research Clinical Research Network and one-to-one discussions with clinicians.

During our internal pilot phase, we identified that recruitment was slower than anticipated. To understand site-level barriers to recruitment, we held three recruitment training meetings with 17 staff members from 7 sites. Feedback from local delivery teams was that patients were willing to participate but our screening processes were complex, and that the workload associated with our recruitment processes was limiting their capacity to recruit patients. Our original recruitment process was to screen patients listed for an MNBB and then recruit patients prior to their MNBB. Patients who had ≥60% pain relief from the MNBB (approximately 40% of patients^45^) were then eligible to proceed in the trial and were listed for RFD and randomised in theatre. This process meant there was a significant time lag (often 18 months or more since the pandemic) between recruitment and randomisation due to NHS waiting lists for MNBB and RFD. Our original pathway also meant that 625 patients needed to be consented into the trial for us to randomise 250. We designed the trial this way to optimise acceptability to patients, as we were concerned that once they are on an established pathway to RFD, they would find randomisation (including the possibility of receiving a placebo) unacceptable. However, the feedback from sites was that patients are willing to participate and are motivated by the desire to help future patients. In particular, they are reassured by a feature we included in the design to promote recruitment, namely the offer of blinded reintervention with the alternative treatment if they do not experience a clinically important improvement in pain after 3 months. In light of this feedback from sites, we simplified our screening and recruitment processes by recruiting patients after they are listed for RFD. This approach substantially reduces the screening and recruitment workload to sites, reduces the time lag between consent and randomisation, and means that we no longer need to consent many more patients than will be randomised.

In summary, our internal pilot phase identified some challenges to trial delivery. We have been proactive in understanding how best to address these challenges and adapting our trial design to optimise delivery.

## supplementary material

10.1136/bmjopen-2023-079173 online supplemental file 1

10.1136/bmjopen-2023-079173 online supplemental file 2
