# Supplementary material for: Effectiveness and cost-effectiveness of radiofrequency denervation versus placebo for chronic and moderate to severe low back pain: study protocol for the RADICAL randomised controlled trial
Source: BMJ Open. 2024 Jul 26;14(7):e079173. doi: 10.1136/bmjopen-2023-079173 (PMC11284913; doi:10.1136/bmjopen-2023-079173)
Supplement: online supplemental file 1 [file bmjopen-14-7-s001.pdf]

Supplementary Information

Mandatory and recommended components of the RFD procedure

Mandatory components include that the numbers and laterality of medial branches to be lesioned should be based on response to the MNBB; lesions to be carried out at 80° Celsius for 90 seconds with two lesions per medial branch, unless a multipronged needle is used (only one lesion required in this case); the position of the RF cannula tip should be adjusted for the second lesion (if required); and x-rays from at least three views should be saved so that needle placement can be evaluated (as required).

Recommended, but not mandatory, components include: a maximum of eight medial branches at a maximum of four vertebral levels lesioned in a single sitting, and participants with unilateral pain to receive unilateral treatment; Chlorhexidine applied for skin preparation, unless the patient is allergic; full aseptic technique used; Lignocaine (local anaesthetic) used for skin infiltration; a curved 18 G RF cannula with a 10mm active tip used for targeting the medial branch (multi-pronged versions permitted); position of RF cannula confirmed with inferior, superior and oblique views; once the needle position is confirmed, optional routine motor testing can be carried out; and local anaesthetic (Lignocaine 20mg/mL in 0.5mL boluses recommended) is infiltrated before the lesion in order to minimise discomfort.

Baseline demographic and medical history

- Sex
- Age
- Body mass index
- Index of multiple deprivation
- Ethnicity
- Employment status
- Smoking status
- E-cigarette user
- Myocardial infarction
- Congestive heart failure
- Peripheral vascular disease
- Cerebrovascular accident
- Transient ischaemic attack
- Dementia

- Chronic obstructive pulmonary disease
- Connective tissue disease
- Peptic ulcer disease
- Hemiplegia
- Liver disease
- Diabetes mellitus
- Moderate to severe chronic kidney disease
- Solid tumour
- Leukaemia
- Lymphoma
- AIDS
- Previous back surgery

TSC and DMSC details

The TSC is made up of representatives from the RADICAL study team and independent members approved by the funder. The DMSC consists of an independent medical statistician and medical experts in this field approved by the funder. The TSC and DMSC meet as frequently as they feel is necessary, usually at least once a year.

Examples of methods used with different Radiofrequency (RF) machines to ensure blinding

Make of the RF machine	Method
Diros	A custom switching box has been developed which allows the unblinded randomiser to switch between 'RFD' and 'placebo' mode, whilst maintaining blinding of the rest of team and the patient in theatre.
Stryker	The beeping noise that is made when a lesion is performed (for RFD) is the same as the beeping noise that is made when sensory mode is on, even at 0.0v. This means that sensory mode at 0.0v can be selected by the randomiser for patients that are allocated to the 'placebo' treatment, and as far as the rest of the team and the patient in theatre are concerned it would sound the same as 'RFD', therefore maintaining blinding.
