# Supplementary material for: Effectiveness and cost-effectiveness of radiofrequency denervation versus placebo for chronic and moderate to severe low back pain: study protocol for the RADICAL randomised controlled trial
Source: BMJ Open. 2024 Jul 26;14(7):e079173. doi: 10.1136/bmjopen-2023-079173 (PMC11284913; doi:10.1136/bmjopen-2023-079173)
Supplement: online supplemental file 2 [file bmjopen-14-7-s002.pdf]

Supplementary Table 1 Schedule of data collection

	Baseline	Randomisation & intervention	Post-randomisation									
			2	4	6	8	10	3	6	12	18	24
			weeks					months				
Sociodemographic details	X											
HADS	X											
Medical history including pain location	X											
STarT Back tool	X											
NRS pain score	X		X	X	X	X	X	X	X	X	X	X
EQ-5D-5L	X				X			X	X	X	X	X
SF-12	X							X	X	X	X	X
ODI	X							X	X	X	X	X
WPAI	X							X	X	X	X	X
Procedural data		X										
Blinded re-intervention offered								X				
Uptake of blinded re-intervention								X	X	X	X	X
Satisfaction with treatment outcome								X	X	X	X	X
Adverse events			X		X			X	X	X	X	X
Resource and health service use questionnaire								X	X	X	X	X
