# Supplementary material for: Effectiveness and cost-effectiveness of radiofrequency denervation versus placebo for chronic and moderate to severe low back pain: study protocol for the RADICAL randomised controlled trial
Source: BMJ Open. 2024 Jul 26;14(7):e079173. doi: 10.1136/bmjopen-2023-079173 (PMC11284913; doi:10.1136/bmjopen-2023-079173)
Supplement: Supplementary file 3 [file bmjopen-2023-079173.reviewer_comments.pdf]

PEER REVIEW HISTORY

BMJ Open publishes all reviews undertaken for accepted manuscripts. Reviewers are asked to complete a checklist review form (**http://bmjopen.bmj.com/site/about/resources/checklist.pdf**) and are provided with free text boxes to elaborate on their assessment. These free text comments are reproduced below.

ARTICLE DETAILS

**TITLE (PROVISIONAL)**	Effectiveness and cost-effectiveness of radiofrequency denervation versus placebo for chronic and moderate to severe low back pain: study protocol for the RADICAL randomised controlled trial
**AUTHORS**	Ashton, Kate; Price, Cathy; Fleming, Leah; Blom, Ashley; Culliford, Lucy; Evans, Rebecca; Foster, Nadine E.; Hollingworth, William; Jameson, Catherine; Jeynes, Nouf; Moore, Andrew; Orpen, Neil; Palmer, Cecily; Reeves, Barnaby; Rogers, Chris; Wylde, Vikki

VERSION 1 – REVIEW

**REVIEWER**	Kallewaard, Jan-Willem<br>Rijnstate, Anesthesiology
**REVIEW RETURNED**	05-Jan-2024

**GENERAL COMMENTS**	very important and well designed study<br><br>i have a few remarks :<br>references :<br>replace ref 1 for a more recent repat study in the lancet in 2023 (burden of low back pain)<br>reference rapm Cohen is important and missing<br>Cohen SP, et al. Reg Anesth Pain Med 2020;45:424–467. doi:10.1136/rapm-2019-101243<br><br>use it to describe an international consensus about the technique<br><br>option:<br>use a recent publication about the evidence a narrative review from 2023 pain pracice: Update of Evidence-based Interventional Pain Medicine<br>according to clinical diagnoses 3. Pain originating from the lumbar facet joints<br><br>pag 12 statistical analysis: do not make the same mistake as was made in the MINT study<br>IMMPact 30 % pain reduction is not enough<br>describe:<br>- which difference bewteween groups is considered statistically significant and what is considered as clinical relavant<br>this is missing but extremely important<br><br>test block: why is choses for 60% pain reduction. i have no problems with 60% but international guidelines agree that 50% is enough<br><br>video is not availabel anymore

	and 40% positive testblocks is more than what is expected form the literature

**REVIEWER**	Maas, Esther<br>Vrije University Amsterdam, Department of Health Sciences
**REVIEW RETURNED**	16-Jan-2024

**GENERAL COMMENTS**	Peer review:<br>Effectiveness and cost-effectiveness of radiofrequency denervation versus placebo for chronic and moderate to severe low back pain: study protocol for the RADICAL randomised controlled trial<br><br>Overall, I would like to complement the authors on this extensive work, and preparation for a randomised controlled trial on an important topic. I am looking forward to the results of the RADICAL trial in a few years.<br><br>I have a few questions for the authors for clarification. I will do this in chronological order of the paper, with minor remarks at the end.<br><br>Major comments:<br>The protocol (Article summary & Methods section) states that the trial has a 'pragmatic design integrated into standard care pathways'. However, reading through the manuscript, the trial seems to balance between an efficacy trial and a pragmatic effectiveness trial. A pragmatic trial is the best vehicle for an economic evaluation, however, choices made in the study design do not fit the purpose of the study. This raises the concern that the clinical care in the trial is not representative of care that occurs in typical practice. The main issues that caused confusion in the positioning of the trial are:<br>1. The choice for a placebo as control group. For a cost-effectiveness evaluation the control arm is supposed to be commonly used in clinical practice (e.g. usual care). In that case, it can be evaluated if the intervention has added value in comparison to usual care, and if it is worth adding radiofrequency denervation to standard care practice. What will be the conclusions from the cost-effectiveness analysis within a placebo-controlled trial, and how will the results be interpreted. Is it an option in the UK to implement a placebo intervention in usual care? In conclusion, what is the rationale for a placebo as control group?<br>2. The treatment variability. In the introduction, the authors state that a previous trial (The MinT trial) received criticism on the variation in the RFD operator protocols. However, this is part of the pragmatic nature of these types of multicenter trials, reflective of common practice. This is reflected in the study set-up of the RADICAL trial as well: Page 16 line 386: Increased flexibility introduced within the RFD procedure protocol to match usual variability in standard practice. Can the authors explain why and how flexibility in the RFD procedure protocol increased, and what the impact of this treatment variability is on the conclusion of the trial results?<br>3. Resulting from the two points stated above: How can the results of the cost-effectiveness analyses be interpreted? If the results are negative, what is the alternative (is this placebo)?<br>4. A difference of 0.84 in the pain severity NRS is selected for the sample size calculation, without further explanation. Can the authors explain why this value was chosen? Do the authors

	consider this an important difference between the groups? Based on previous literature [1], a minimal clinical important difference of 2 out of 10 on the NRS has been identified. Furthermore, the flowchart also states: "Blinded re-intervention with alternative treatment offered to patients who improve <2 points on pain NRS at 3 months. This suggests the awareness and importance of at least a minimal important change of 2 points. Can the authors clarify this choice, and explain how statistically significant results can be interpreted (are these also clinically relevant?)?<br>[1] Ostelo RW, Deyo RA, Stratford P, et al. Interpreting change scores for pain and functional status in low back pain: towards international consensus regarding minimal important change. Spine (Phila Pa 1976) 2008;33(1):90-4. doi: 10.1097/BRS.0b013e31815e3a10<br>5. The protocol states that the radiofrequency machines 'will have to meet key criteria' without specifying the required criteria for safeguarding the double blinding. Secondly, that authors state that as a change in the protocol muting the sound of the RF machine was found not to be an option due to safety factors, and sites must have an alternative appropriate solution in place. Can the authors elaborate on the blinding procedure, and specifically key criteria for blinding, and the alternative solutions that the sites had in place? Can blinding be guaranteed?<br><br>Minor comments<br>1. The rationale of a 2-year follow-up remains unclear. To what extent can the (reduction in) low back pain be attributed to the RF, two years after a single intervention?<br>2. What is the rationale for the inclusion of patients with moderate LBP? Previous studies have usually only included patients with severe low back pain (NRS >=7). By including patients with moderate low back pain, there's a smaller range for improvement. Can the authors explain if this is related to the choice of the 0.84 as minimal important difference?<br>3. For the subgroup analysis, a cutoff is made for reduction in NRS scores, with one group having a >=80% reduction, and the second group having a >=60-79% reduction. What is the rationale for these cut-off scores? And does this mean all participants with <60% reduction are excluded from secondary analysis? If this is the case, what is the rationale for this exclusion?<br>4. No statement or inclusion criteria is provided regarding co-interventions of medication use of participants while participating in the trial. Can the authors elaborate on this? Are patients allowed to use medication (opioids, or NSAIDS for example) or go to a primary care practitioner in the duration of the trial follow-up period.<br>5. The data collection section only states that 'some demographic data' will be collected and presenting only three examples. Can the authors provide a full list of collected demographic data.<br>6. The methods for imputation are minimally described. Can the authors mention the method of imputation (e.g. multiple imputation), including a reference?<br>7. For the economic evaluation, different perspectives are mentioned in the abstract, but are not clearly stated in the cost-effectiveness analyses section. One of these perspectives is the social service perspective, but if this is truly one of the selected perspectives, why is absenteeism (a measure for the societal perspective) measured? And if it should be a societal perspective (as well), why is presenteeism not measured?

	8. Table 1 illustrates Psychological Distress will be measured at baseline, but this variable is not measured elsewhere in the protocol. How is this operationalized and measured?<br>9. Pay attention to abbreviations, as not every abbreviation is explained in the text.<br>10. A Data availability/open access statement is missing<br>11. The statistical analysis plan, health economic analysis plan, and the data management plan are missing from the protocol<br><br>I would like to thank the authors and the editor for letting me review this manuscript, and I'm looking forward to the author's reply.

VERSION 1 – AUTHOR RESPONSE

Reviewer: 1

references :
replace ref 1 for a more recent repat study in the lancet in 2023 (burden of low back pain)
reference rapm Cohen is important and missing
Cohen SP, et al. Reg Anesth Pain Med 2020;45:424–467. doi:10.1136/rapm-2019-101243

use it to describe an international consensus about the technique
Thank you for these helpful suggestions, we have now changed reference 1 to the 2023 Lancet article.

We have amended our intervention section in the methods to explain that there are international practice guidelines for performing RFD (with reference to the Cohen et al article), however because of the variation in RFD technique across the UK, we held a national consensus workshop to refine the RFD technique for the trial (lines 180-183, page 9).
use a recent publication about the evidence a narrative review from 2023 pain practice: Update of Evidence-based Interventional Pain Medicine
according to clinical diagnoses 3. Pain originating from the
lumbar facet joints
Thank you, we have now added this reference to the introduction section (line 97, page 6).
page 12 statistical analysis: do not make the same mistake as was made in the MINT study
IMMPact 30 % pain reduction is not enough
describe:
- which difference between groups is considered statistically significant and what is considered as clinical relevant
this is missing but extremely important The RADICAL trial is powered to detect a difference of at least 0.84 in the pain severity numerical rating scale (scored 0-10) between randomised groups with 90% power and 5% 2-tailed significance. The National Institute of Clinical Excellence (NICE) uses 0.5 SD as an estimate of a clinically significant between group change (i.e. a NRS difference of 1). This gives a 0.5 effect size (moderate). This is the target difference used by NICE and is sufficient to lead to changes in UK practice. We are powered to detect a difference of 0.84 to account for uncertainty. This is the minimum difference we are powered to detect, and the trial will be able to detect larger differences in pain severity between randomised groups if they exist. We have clarified this in the manuscript by adding the following sentence to the sample size section:

"The trial will therefore have sufficient power to detect the target difference used by NICE (1-point difference) and reflects a moderate effect size."

test block: why is choses for 60% pain reduction. i have no problems with 60% but international guidelines agree that 50% is enough
This is based on our national consensus workshop with clinicians, in which there was a strong preference for the threshold for defining a positive response to MNBB be higher than 50% to ensure that patients most likely to benefit from RFD were selected for the RADICAL trial. Setting the threshold at ⩾80% was discussed; however, review of the MINT-aggregated data showed that this would greatly reduce the number of patients eligible for the trial. Therefore, consensus was reached that setting the threshold at 60% would facilitate appropriate patient selection and trial delivery. This is described and discussed in our published article from the consensus workshops. Due to restrictions on word count we are unable to provide full details in this protocol paper, however we have referred readers to our previous article by providing a reference in the eligibility section of the methods.
video is not available anymore
The video is hosted on Youtube and can be accessed via this link https://www.youtube.com/watch?v=j4nzkdgMWgI.

We have checked and the link is working, and the video is live and set to be publicly accessible, therefore we are unsure as to why the video was not available for you.
and 40% positive testblocks is more than what is expected form the literature
We estimated a 40% response based on the published literature and we have now added a reference to the discussion (line 424, page 18).

Reviewer: 2

Major comments
The protocol (Article summary & Methods section) states that the trial has a 'pragmatic design integrated into standard care pathways'. However, reading through the manuscript, the trial seems to balance between an efficacy trial and a pragmatic effectiveness trial. A pragmatic trial is the best vehicle for an economic evaluation, however, choices made in the study design do not fit the purpose of the study. This raises the concern that the clinical care in the trial is not representative of care that occurs in typical practice. The main issues that caused confusion in the positioning of the trial are:

1. The choice for a placebo as control group. For a cost-effectiveness evaluation the control arm is supposed to be commonly used in clinical practice (e.g. usual care). In that case, it can be evaluated if the intervention has added value in comparison to usual care, and if it is worth adding radiofrequency denervation to standard care practice. What will be the conclusions from the cost-effectiveness analysis within a placebo-controlled trial, and how will the results be interpreted. Is it an option in the UK to implement a placebo intervention in usual care? In conclusion, what is the rationale for a placebo as control group?
This trial was commissioned and funded by the UK National Institute for Health Research Health Technology Assessment (NIHR HTA) panel and the use of a placebo was mandated in the commissioning brief that was released by the NIHR HTA. A placebo control is important in this trial to minimise bias as our primary outcome is a patient-reported measure of pain at 3 months after randomisation. A placebo control does not necessary imply that a trial is not pragmatic, the control is to achieve absence of bias since our primary outcome is patient-reported.

We have clarified this in the description of the placebo in the methods by added “A placebo control will be used to minimise bias, which is important as the primary outcome is patient-reported" (lines 188-189, page 10).

Cost effectiveness of the actual RFD procedure can be assessed even in a trial that uses placebo.

2. The treatment variability. In the introduction, the authors state that a previous trial (The MinT trial) received criticism on the variation in the RFD operator protocols. However, this is part of the pragmatic nature of these types of multicenter trials, reflective of common practice. This is reflected in the study set-up of the RADICAL trial as well: Page 16 line 386: Increased flexibility introduced within the RFD procedure protocol to match usual variability in standard practice. Can the authors explain why and how flexibility in the RFD procedure protocol increased, and what the impact of this treatment variability is on the conclusion of the trial results?
The changes made allowed increased flexibility in the RFD clinical protocol and involved splitting items within the RFD protocol into 'mandatory' and 'recommended' (see below for explanation). Some of the items were also updated to reflect advances in equipment (e.g. use of multipronged needles negating the need for 2 lesions per medial branch).

To develop the consensus-based RFD clinical protocol, we used a published framework for the standardisation and delivery of surgical interventions within RCTs, which is increasing being adopted for surgical trials in the UK (https://pubmed.ncbi.nlm.nih.gov/27462835). This approach describes each step of a surgical procedure as prohibited, mandatory or optional. Previously, the study protocol implied that all of the steps were mandatory. The changes were made to clarify which of the steps were in fact mandatory, and which were 'recommended', but optional. These changes were made to ensure that the RFD technique reflects how RFD is performed in the NHS, thereby facilitating the implementation and uptake of the trial findings if RFD is shown to be effective, while adhering to our consensus-based best practice recommendations developed in our national workshop.

We have amended the wording in the manuscript to make this clearer (lines 377-378, page 17).

3. Resulting from the two points stated above: How can the results of the cost-effectiveness analyses be interpreted? If the results are negative, what is the alternative (is this placebo)?
If the trial shows that RFD is not clinically or cost-effective compared to the placebo, then we would not recommend that clinicians use RFD in practice. We would also not recommend that patients are offered placebo, the placebo is there to ensure any differences observed can be attributed to the intervention.

4. A difference of 0.84 in the pain severity NRS is selected for the sample size calculation, without further explanation. Can the authors explain why this value was chosen? Do the authors consider this an important difference between the groups? Based on previous literature [1], a minimal clinical important difference of 2 out of 10 on the NRS has been identified. Furthermore, the flowchart also states: "Blinded re-intervention with alternative treatment offered to patients who improve <2 points on pain NRS at 3 months. This suggests the awareness and importance of at least a minimal important change of 2 points. Can the authors clarify this choice, and explain how statistically significant results can be interpreted (are these also clinically relevant?)?
[1] Ostelo RW, Deyo RA, Stratford P, et al. Interpreting change scores for pain and functional status in low back pain: towards international consensus regarding minimal important change. Spine (Phila Pa 1976) 2008;33(1):90-4. doi: 10.1097/BRS.0b013e31815e3a10
Thank you for highlighting this, which was also raised by Reviewer 1. The RADICAL trial is powered to detect a difference of at least 0.84 in the pain severity numerical rating scale (scored 0-10) between randomised groups with 90% power and 5% 2-tailed significance. The National Institute of Clinical Excellence (NICE) uses 0.5 SD as an estimate of a clinically significant between group change (i.e. a NRS difference of 1). This gives a 0.5 effect size (moderate). This is the target difference used by NICE and is sufficient to lead to changes in UK practice. We are powered to detect a difference of 0.84 to account for uncertainty. This is the minimum difference we are powered to detect, and the trial

will be able to detect larger differences in pain severity between randomised groups if they exist. We have clarified this in the manuscript by adding the following sentence to the sample size section:

“The trial will therefore have sufficient power to detect the target difference used by NICE (1-point difference) and reflects a moderate effect size.”

5. The protocol states that the radiofrequency machines ‘will have to meet key criteria’ without specifying the required criteria for safeguarding the double blinding. Secondly, that authors state that as a change in the protocol muting the sound of the RF machine was found not to be an option due to safety factors, and sites must have an alternative appropriate solution in place. Can the authors elaborate on the blinding procedure, and specifically key criteria for blinding, and the alternative solutions that the sites had in place? Can blinding be guaranteed?
As the method employed varies by the make of the RF machine, we have now added examples of the methods used with different RF machines to ensure blinding. These have been added to the Supplementary file.

We are using the Bang Blinding Index to assess the success of blinding; this is described on line 177, page 9.

Minor comments
1. The rationale of a 2-year follow-up remains unclear. To what extent can the (reduction in) low back pain be attributed to the RF, two years after a single intervention?
The duration of pain relief following radiofrequency denervation is uncertain. Data from randomised controlled trials suggests relief is maintained for at least 6-12 months but no study has reported longer term outcomes. Pain relief for more than two years would not be an unreasonable clinical expectation. Because of this, a minimum duration of two years follow-up was mandated in the NIHR HTA commissioning brief for this trial.
2. What is the rationale for the inclusion of patients with moderate LBP? Previous studies have usually only included patients with severe low back pain (NRS >=7). By including patients with moderate low back pain, there’s a smaller range for improvement. Can the authors explain if this is related to the choice of the 0.84 as minimal important difference?
We are including patients with moderate-severe LBP to reflect UK national clinical guidelines. The NICE guidance for low back pain and sciatica (NG59) states that RFD should be considered for patients who meet the diagnostic criteria and have moderate or severe levels of localised back pain (rated as 5 or more on a visual analogue scale, or equivalent).

3. For the subgroup analysis, a cutoff is made for reduction in NRS scores, with one group having a >=80% reduction, and the second group having a >=60-79% reduction. What is the rationale for these cut-off scores? And does this mean all participants with <60% reduction are excluded from secondary analysis? If this is the case, what is the rationale for this exclusion?
The subgroup analysis is based on response to the MNBB, rather than RFD. To be eligible for the trial, all patients must have had a response of ≥60% to the MNBB. We plan to do a subgroup analysis based on MNBB response as there were some pain clinicians at our consensus workshop that set the threshold for RFD at ≥80% pain relief from the MNBB; therefore, we will do a subgroup analysis of the primary outcome based on response to MNBB.

4. No statement or inclusion criteria is provided regarding co-interventions of medication use of participants while participating in the trial. Can the authors elaborate on this? Are patients allowed to use medication (opioids, or NSAIDS for example) or go to a primary care practitioner in the duration of the trial follow-up period.

We did not place any restrictions on usual care, and all co-interventions were permitted to reflect usual NHS practice. This has been clarified in the manuscript (lines 148-149, page 8).
5. The data collection section only states that ‘some demographic data’ will be collected and presenting only three examples. Can the authors provide a full list of collected demographic data. We have now added a full list of the demographic data collected to the supplementary file.
6. The methods for imputation are minimally described. Can the authors mention the method of imputation (e.g. multiple imputation), including a reference?
We have added further details about the imputation methods to be used in the statistical analyses (lines 277-280, page 13) and the health economic analyses (line 307, page 14). We will provide more information in a separate analysis plan.

7. For the economic evaluation, different perspectives are mentioned in the abstract, but are not clearly stated in the cost-effectiveness analyses section. One of these perspectives is the social service perspective, but if this is truly one of the selected perspectives, why is absenteeism (a measure for the societal perspective) measured? And if it should be a societal perspective (as well), why is presenteeism not measured?
The NHS and social service perspective is the primary perspective as it is the one often used by NICE in the UK. In sensitivity analysis, we broaden the perspective to include all of presenteeism, absenteeism (using the WPAI) and informal care.
8. Table 1 illustrates Psychological Distress will be measured at baseline, but this variable is not measured elsewhere in the protocol. How is this operationalized and measured?
We have now clarified in Table 1 that the psychological distress variable at baseline is being measured using the STarT Back tool. Mental health is being measured using the SF-12 MCS subscale at baseline, 3 months, 6 months, 12 months 18 months and 24 months.
9. Pay attention to abbreviations, as not every abbreviation is explained in the text.
Thank you for highlight this, we have now reviewed the manuscript and explained all abbreviations.
10. A Data availability/open access statement is missing
Our data availability statement is included in the ‘data handling, storage and sharing section’ and states:

“In compliance with the Medical Research Council Policy on Data Sharing, and with participant agreement, relevant ‘meta’-data about the trial and the full dataset, but without any participant identifiers other than the unique study identifier, will be held indefinitely. These will be retained because of the potential for the raw data to be used subsequently for secondary research and/or training.”

11. The statistical analysis plan, health economic analysis plan, and the data management plan are missing from the protocol We are preparing a separate Statistical Analysis plan and Health Economics Analysis plan, and we will make these publicly available through the University of Bristol website before the database is locked for analysis, which reflects standard practice for clinical trials in the UK.

In addition to the changes requested by the reviewers, we have made some small updates to the discussion to reflect the current number of sites open, and the number of patients recruited and randomised. We have also edited the randomisation and blinding section to make the distinction between the clinician performing the RFD procedure (the ‘operator’) and the person randomising (the ‘randomiser’) clearer.

VERSION 2 – REVIEW

**REVIEWER**	Maas, Esther

	Vrije University Amsterdam, Department of Health Sciences
**REVIEW RETURNED**	05-Mar-2024

**GENERAL COMMENTS**	I thank the authors for their careful consideration of the comments and questions in the first peer-review. I understand that the authors were not able to make all changes as suggested because this is a protocol paper for an ongoing and commissioned trial.<br>I have a few minor last comments/questions/reflections:<br><br>Reflection on response to major comments:<br>Comment 1. A placebo-controlled trial was commissioned and funded by the UK National Institute for Health Research Health Technology Assessment (NIHR HTA). This is unchangeable; however, the framing of a pragmatic trial could be re-evaluated. In pragmatic randomized controlled trials, participants should be similar to those who will experience the intervention if it is implemented broadly, monitoring should be done under routine circumstances, and interventions should be compared with usual practice1. This is important to reflect upon when describing and interpreting the results of this trial.<br>https://www.ncbi.nlm.nih.gov/pmc/articles/PMC4047300/<br>Comment 4. Can you add a reference to the NICE guideline when referring to the 1-point difference that reflects a moderate effect size.<br><br>Reflection on response to minor comments:<br>Comment 1. It is understandable that the 2-year follow-up was mandated. However, it remains questionable how much of the effect can be attributed to the RF-denervation 2 years after treatment. This should be reflected upon when publishing these results.<br>Comment 4. All cointerventions are permitted, but are they also measured, and reported?

VERSION 2 – AUTHOR RESPONSE

Reviewer: 2

Reflection on response to major comments:

Comment 1. A placebo-controlled trial was commissioned and funded by the UK National Institute for Health Research Health Technology Assessment (NIHR HTA). This is unchangeable; however, the framing of a pragmatic trial could be re-evaluated. In pragmatic randomized controlled trials, participants should be similar to those who will experience the intervention if it is implemented broadly, monitoring should be done under routine circumstances, and interventions should be compared with usual practice1. This is important to reflect upon when describing and interpreting the results of this trial.

https://www.ncbi.nlm.nih.gov/pmc/articles/PMC4047300/

Thank you for this helpful advice which we will reflect upon in our publication of the trial findings.

Comment 4. Can you add a reference to the NICE guideline when referring to the 1-point difference that reflects a moderate effect size.

Apologies for this oversight, we have added a reference now (page 12).

Reflection on response to minor comments:

Comment 1. It is understandable that the 2-year follow-up was mandated. However, it remains questionable how much of the effect can be attributed to the RF-denervation 2 years after treatment. This should be reflected upon when publishing these results.

Thank you, we will include discussion of this in the publication of our trial findings.

Comment 4. All cointerventions are permitted, but are they also measured, and reported?

Data on co-interventions are not being collected. We have clarified this in the manuscript (page 8).
